# Supplementary material for: Gender differences in earnings among people with multiple sclerosis and associations with type of occupation and family composition: A population-based study with matched references
Source: PLoS One. 2023 Aug 2;18(8):e0288998. doi: 10.1371/journal.pone.0288998 (PMC10395842; doi:10.1371/journal.pone.0288998)
Supplement: S1 Fig — (PDF) [file pone.0288998.s001.pdf]

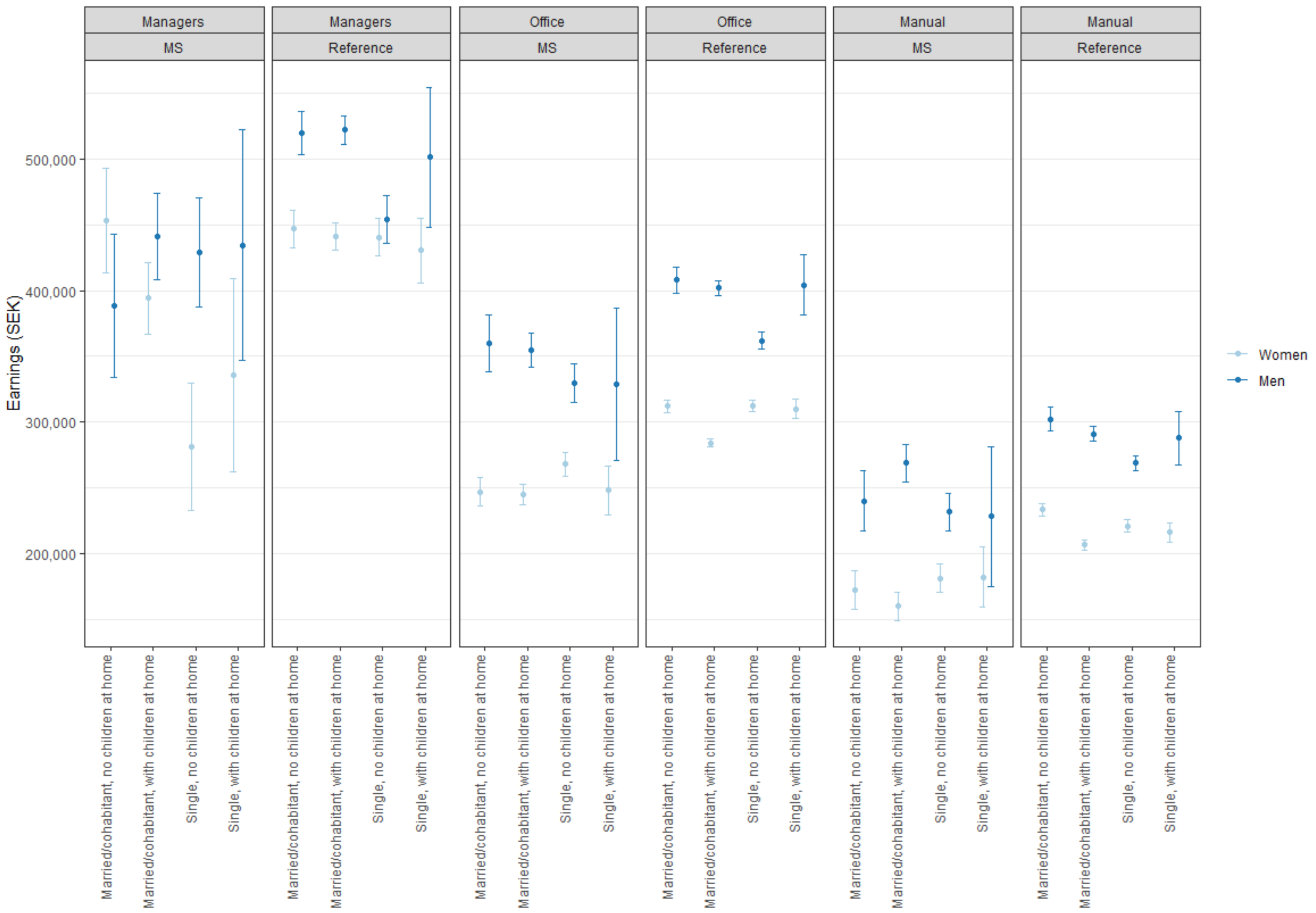

Supplementary Figure 1. Four-way weighted least-squares analysis of covariance (WLS ANCOVA) plots for mean earnings (in Swedish Krona, SEK) between gender (women, in light blue vs men, in dark blue), presence of MS or not (MS vs references), occupation (managerial, office, or manual workers), and family composition (married/cohabitant or single, and with or without children <18 years at home, respectively) when mutually adjusted and controlling for age and type of living area only.
