# Supplementary material for: Gender differences in earnings among people with multiple sclerosis and associations with type of occupation and family composition: A population-based study with matched references
Source: PLoS One. 2023 Aug 2;18(8):e0288998. doi: 10.1371/journal.pone.0288998 (PMC10395842; doi:10.1371/journal.pone.0288998)
Supplement: S1 Table — Abbreviations: MS, Multiple sclerosis; Sd, Standard deviation; SEK, Swedish Krona; SA, Sickness absence; DP, disability pension. Both cohorts were matched on age, gender, type of living area, and county with a ratio of 5 reference individuals sampled from the general population to one person with multiple sclerosis (MS). (PDF) [file pone.0288998.s002.pdf]

Supplementary Table 1. Sociodemographic, economic, and clinical characteristics in 2009 of people with multiple sclerosis (PwMS) and of matched references, by gender and among all

	People with MS			Reference group (No MS)		
**Gender**	**Women**	**Men**	**All with MS**	**Women**	**Men**	**All references**
	n=5902	n=2295	n=8197	n=29510	n=11475	n=40985
	72.0%	28.0%	100%	72.0%	28.0%	100%
**Age**						
19-24	194 (3.3)	70 (3.1)	264 (3.2)	970 (3.3)	350 (3.1)	1320 (3.2)
25-34	1046 (17.7)	397 (17.3)	1443 (17.6)	5230 (17.7)	1985 (17.3)	7215 (17.6)
35-44	1933 (32.8)	795 (34.6)	2728 (33.3)	9665 (32.8)	3975 (34.6)	13640 (33.3)
45-54	2147 (36.4)	803 (35.0)	2950 (36.0)	10735 (36.4)	4015 (35.0)	14750 (36.0)
55-57	582 (9.9)	230 (10.0)	812 (9.9)	2910 (9.9)	1150 (10.0)	5060 (9.9)
Mean age (sd)	42.7 (9.2)	42.6 (9.2)	42.6 (9.2)	42.7 (9.2)	42.6 (9.2)	42.6 (9.2)
**Educational level**						
Compulsory school <10 years	539 (9.1)	340 (14.8)	879 (10.7)	3468 (11.8)	1892 (16.5)	5360 (13.1)
Upper secondary school 10–12 years	2837 (48.1)	1183 (51.5)	4019 (49.0)	13432 (45.5)	5728 (49.9)	19160 (46.7)
University/college >12 years	2526 (42.8)	773 (33.7)	3299 (40.2)	12610 (42.7)	3855 (33.6)	16465 (40.2)
**Born in Sweden**						
Yes	5341 (90.5)	2039 (88.8)	7380 (90.0)	23994 (81.3)	9423 (82.1)	33417 (81.5)
No	561 (9.5)	256 (11.2)	817 (10.0)	5516 (18.7)	2052 (17.9)	7568 (18.5)
**Type of living area**						
Larger cities	2306 (39.1)	940 (41.0)	3246 (39.6)	11540 (39.1)	4700 (41.0)	16230 (39.6)
Medium-sized towns	2043 (34.6)	789 (34.4)	2832 (34.5)	10215 (34.6)	3945 (34.4)	14160 (34.5)
Small towns	1553 (26.3)	566 (24.7)	2119 (25.9)	7765 (26.3)	2830 (24.7)	10595 (25.9)
**County**						
Stockholm	1336 (22.6)	504 (22.0)	1840 (22.4)	6680 (22.6)	2520 (22.0)	9200 (22.4)
Uppsala	245 (4.2)	89 (3.9)	334 (4.1)	1225 (4.2)	445 (3.9)	1670 (4.1)
Södermanland	154 (2.6)	60 (2.6)	214 (2.6)	770 (2.6)	300 (2.6)	1070 (2.6)
Östergötland	311 (5.3)	144 (6.3)	455 (5.6)	1555 (5.3)	720 (6.3)	2275 (5.6)
Jönköping	199 (3.4)	76 (3.3)	275 (3.4)	995 (3.4)	380 (3.3)	1375 (3.4)
Kronoberg	87 (1.5)	26 (1.1)	113 (1.4)	435 (1.5)	130 (1.1)	565 (1.4)
Kalmar	107 (1.8)	58 (2.5)	165 (2.0)	535 (1.8)	290 (2.5)	825 (2.0)
Gotland	41 (0.7)	14 (0.6)	55 (0.7)	205 (0.7)	70 (0.6)	275 (0.7)
Blekinge	97 (1.6)	26 (1.1)	123 (1.5)	485 (1.6)	130 (1.1)	615 (1.5)
Skåne	684 (11.6)	288 (12.5)	972 (11.9)	3420 (11.6)	1440 (12.5)	4860 (11.9)
Halland	175 (3.0)	78 (3.4)	253 (3.1)	875 (3.0)	390 (3.4)	1265 (3.1)
Västra Götaland	1015 (17.2)	419 (18.3)	1434 (17.5)	5075 (17.2)	2095 (18.3)	7170 (17.5)
Värmland	235 (4.0)	79 (3.4)	314 (3.8)	1175 (4.0)	395 (3.4)	1570 (3.8)

Örebro	191 (3.2)	52 (2.3)	243 (3.0)	955 (3.2)	260 (2.3)	1215 (3.0)
Västmanland	125 (2.1)	49 (2.1)	174 (2.1)	625 (2.1)	245 (2.1)	870 (2.1)
Dalarna	211 (3.6)	56 (2.4)	267 (3.3)	1055 (3.6)	280 (2.4)	1335 (3.3)
Gävleborg	164 (2.8)	63 (2.7)	227 (2.8)	820 (2.8)	315 (2.7)	1135 (2.8)
Västernorrland	128 (2.2)	47 (2.0)	175 (2.1)	640 (2.2)	235 (2.0)	875 (2.1)
Jämtland	92 (1.6)	41 (1.8)	133 (1.6)	460 (1.6)	205 (1.8)	665 (1.6)
Västerbotten	186 (3.2)	85 (3.7)	271 (3.3)	930 (3.2)	425 (3.7)	1355 (3.3)
Norrbotten	119 (2.0)	41 (1.8)	160 (2.0)	595 (2.0)	205 (1.8)	800 (2.0)
**Family composition**						
Married/cohabitant, no children at home	1292 (21.9)	386 (16.8)	1678 (20.5)	5939 (20.1)	1666 (14.5)	7605 (18.6)
Married/cohabitant, with children at home	2067 (35.0)	815 (35.5)	2882 (35.2)	11 755 (39.8)	4683 (40.8)	16 438 (40.1)
Single, no children at home	2074 (35.1)	1032 (45.5)	3106 (37.9)	8884 (30.1)	4821 (42.0)	13 705 (33.4)
Single, with children at home	469 (7.9)	61 (2.7)	531 (6.5)	2932 (9.9)	305 (2.7)	3237 (7.9)
**Employment status**						
In paid work	3860 (65.4)	1579 (68.8)	5439 (66.4)	23 425 (79.4)	9423 (82.1)	38 848 (80.1)
Not in paid work	2042 (34.6)	716 (31.2)	2758 (33.6)	6084 (20.6)	2052 (17.9)	8137 (19.9)
**Occupation**						
Managers	147 (2.5)	117 (5.1)	264 (3.2)	1144 (3.9)	888 (7.7)	2032 (5.0)
Office	2487 (42.1)	785 (34.2)	3272 (39.9)	12495 (42.3)	3891 (33.9)	16 386 (40.0)
Manual	1614 (27.3)	775 (33.8)	2389 (29.1)	10861 (36.8)	4753 (41.4)	15 614 (38.1)
Not identified	157 (2.7)	78 (3.4)	235 (2.9)	987 (3.3)	544 (4.7)	1531 (3.7)
Not in work	1497 (25.4)	533 (23.3)	2030 (24.8)	4014 (13.6)	1355 (11.8)	5369 (13.1)
**Earnings from work (SEK)**						
Mean gross income (sd)	151 489 (159 163.8)	208 081 (215 558.2)	167 334 (178 579.1)	210 732 (165 924.7)	287 841 (267 520.7)	232 321 (202 626.5)
**SA/DP net days (2009)**						
Mean SA/DP net days (sd)	228.4 (135.0)	230.1 (144.3)	229.2 (143.3)	228.2 (142.1)	233.9 (148.8)	228.8 (137.43)
**MS disease duration**						
Mean year (sd)	8.8 (7.2)	8.3 (7.0)	8.7 (7.2)	-	-	-

Abbreviations: MS, Multiple sclerosis; Sd, Standard deviation; SEK, Swedish Krona; SA, Sickness absence; DP, disability pension.
Both cohorts were matched on age, gender, type of living area, and county with a ratio of 5 reference individuals sampled from the general population to one person with multiple sclerosis (MS).
