# Supplementary material for: Gender differences in earnings among people with multiple sclerosis and associations with type of occupation and family composition: A population-based study with matched references
Source: PLoS One. 2023 Aug 2;18(8):e0288998. doi: 10.1371/journal.pone.0288998 (PMC10395842; doi:10.1371/journal.pone.0288998)
Supplement: S2 Table — Abbreviations: CI, Confidence interval; p: p values. Significant results are marked in bold (p<0.05). (PDF) [file pone.0288998.s003.pdf]

Supplementary Table 2. Regression Estimates (Robust (weighted) regression, with heteroscasticity corrected (HC3) standard errors/confidence intervals)

	Earnings from work 2010	
Predictors	*Estimates (95% CI)*	*p*
(Intercept)	308258 (293526 – 322979)	**<0.001**
**Age**	30145 (2877 – 3152)	**<0.001**
**Type of living area**		
Big cities	—	
Medium-sized towns	-21210 (-23723 – -18698)	**<0.001**
Small towns	-28514 (-31281 – -25746)	**<0.001**
**Combined SA&DP net days in 2009**	-199 (-249 – -149)	**<0.001**
**Combined SA&DP net days in 2010**	-610 (-658 – -562)	**<0.001**
**Number of children <18 years at home**	-911 (-2933 – 1111)	0.377
**MS**		
Reference (No MS)	—	
PwMS	26701 (-1086 – 64263)	0.164
**Family composition**		
Married/cohabitant, no children at home	—	
Married/cohabitant, with children at home	17779 (1618 – 33939)	**0.031**
Single, no children at home	6826 (-11142 – 24793)	0.457
Single, with children at home	4987 (-20839 – 30813)	0.705
**Occupation**		
Manager	—	
Office	-127780 (-141323 – -114238)	**<0.001**
Manual	-197054 (-210707 – -183401)	**<0.001**
**Gender**		
Women	—	
Men	67638 (48314 – 86963)	**<0.001**
**MS * Family composition**		
PwMS * Married/cohabitant, with children at home	-42400 (-87928 – 3128)	0.068
PwMS * Single, no children at home	-122688 (-181056 – -64320)	**<0.001**
PwMS * Single, with children at home	-101882 (-180208 – -23556)	**0.011**
**MS * Occupation**		
PwMS * Office	-24232 (-63252 – 14789)	0.224
PwMS * Manual	-14034 (-54036 – 25968)	0.492
**Family composition * Occupation**		
Married/cohabitant, with children at home * Office	-19771 (-36316 – -3227)	**0.019**
Single, no children at home * Office	14338 (-4459 – 33136)	0.135
Single, with children at home * Office	10925 (-15879 – 37729)	0.424
Married/cohabitant, with children at home * Manual	-184960 (-35245 – -1745)	**0.030**
Single, no children at home * Manual	4805 (-14105 – 23714)	0.618
Single, with children at home * Manual	218 (-26588 – 27023)	0.987
**MS * Gender**		
PwMS * Men	-109151 (-172151 – -46150)	**0.001**
**Family composition * Gender**		
Married/cohabitant, with children at home * Men	9726 (-13590 – 33042)	0.414
Single, no children at home * Men	-45590 (-73628 – -17552)	**0.001**
Single, with children at home * Men	9477 (-46140 – 65095)	0.738
**Occupation* Gender**		
Office * Men	23241 (1648 – 44834)	**0.035**
Manual * Men	-7460 (-28894 – 13975)	0.495
**MS * Family composition * Occupation**		
PwMS * Married/cohabitant, with children at home * Office	47164 (-122 – 94449)	0.051
PwMS * Single, no children at home * Office	120724 (60773 – 180675)	**<0.001**
PwMS * Single, with children at home * Office	92273 (11238 – 173309)	**0.026**
PwMS * Married/cohabitant, with children at home * Manual	46427 (-2140 – 94994)	0.061
PwMS * Single, no children at home * Manual	117165 (56277 – 178052)	**<0.001**
PwMS * Single, with children at home * Manual	115636 (33327 – 197945)	**0.006**

**MS * Family composition * Gender**		
PwMS * Married/cohabitant, with children at home * Men	62012 (-12588 – 136612)	0.103
PwMS * Single, no children at home * Men	211441 (124334 – 298548)	**<0.001**
PwMS * Single, with children at home * Men	163229 (32980 – 293478)	**0.014**
**MS * Occupation* Gender**		
PwMS * Office * Men	88930 (21599 – 156262)	**0.010**
PwMS * Manual * Men	96177 (28078 – 164275)	**0.006**
**Family composition * Occupation* Gender**		
Married/cohabitant, with children at home * Office * Men	13120 (-12709 – 38948)	0.319
Single, no children at home * Office * Men	5172 (-25266 – 35609)	0.739
Single, with children at home * Office * Men	-11070 (-71471 – 49331)	0.719
Married/cohabitant, with children at home * Manual * Men	8755 (-17042 – 34551)	0.506
Single, no children at home * Manual * Men	27938 (-2224 – 58100)	0.069
Single, with children at home * Manual * Men	-6489 (-66071 – 53094)	0.831
**MS * Family composition * Occupation* Gender**		
PwMS * Married/cohabitant, with children at home * Office * Men	-80363 (-159970 – -757)	**0.048**
PwMS * Single, no children at home * Office * Men	-194363 (-286164 – -102562)	**<0.001**
PwMS * Single, with children at home * Office * Men	-166467 (-311089 – -21846)	**0.024**
PwMS * Married/cohabitant, with children at home * Manual * Men	-50830 (-131546 – 29886)	0.217
PwMS * Single, no children at home * Manual * Men	-193018 (-285454 – -100583)	**<0.001**
PwMS * Single, with children at home * Manual * Men	-188489 (-332165 – -44812)	**0.010**

Abbreviations: CI, Confidence interval; p: p values.
Significant results are marked in bold (p<0.05).
