# Supplementary material for: Gender differences in earnings among people with multiple sclerosis and associations with type of occupation and family composition: A population-based study with matched references
Source: PLoS One. 2023 Aug 2;18(8):e0288998. doi: 10.1371/journal.pone.0288998 (PMC10395842; doi:10.1371/journal.pone.0288998)
Supplement: Attachment — Submitted filename: Response to reviewers R1.docx [file pone.0288998.s004.docx]

**RESPONSE LETTER**

Manuscript PONE-D-22-32848

Dear Editor,

Thank you for the possibility to submit a revised version of our manuscript titled "**Gender differences in earnings among people with multiple sclerosis and associations with type of occupation and family composition: a population-based study with matched references**". We also thank the reviewer for the valuable comments. We have carefully addressed the issue raised. Please, find below our point-by-point responses. In addition, changes have been made according to the comments and tracked in the revised manuscript, including reference list formating, using the Track-Changes tool.

On behalf of all authors, yours sincerely,

Alejandra Machado,
Corresponding author

**Reviewer Comments**

**Reviewer's Responses to Questions**

1. Is the manuscript technically sound, and do the data support the conclusions?

The manuscript must describe a technically sound piece of scientific research with data that supports the conclusions. Experiments must have been conducted rigorously, with appropriate controls, replication, and sample sizes. The conclusions must be drawn appropriately based on the data presented.

Reviewer #1: Yes

________________________________________

2. Has the statistical analysis been performed appropriately and rigorously?

Reviewer #1: Yes

________________________________________

3. Have the authors made all data underlying the findings in their manuscript fully available?

The PLOS Data policy requires authors to make all data underlying the findings described in their manuscript fully available without restriction, with rare exception (please refer to the Data Availability Statement in the manuscript PDF file). The data should be provided as part of the manuscript or its supporting information, or deposited to a public repository. For example, in addition to summary statistics, the data points behind means, medians and variance measures should be available. If there are restrictions on publicly sharing data—e.g. participant privacy or use of data from a third party—those must be specified.

Reviewer #1: Yes

________________________________________

4. Is the manuscript presented in an intelligible fashion and written in standard English?

PLOS ONE does not copyedit accepted manuscripts, so the language in submitted articles must be clear, correct, and unambiguous. Any typographical or grammatical errors should be corrected at revision, so please note any specific errors here.

Reviewer #1: Yes

**Comments to the Author**

**Reviewer #1:** Thank you to the authors for this interesting piece of research. This paper aimed to determine the relationship between earnings and gender for those diagnosed with MS. The analysis also analysed the interactions between gender, MS occupation, family status. Comparisons were made between those without MS across occupation type and family status. The study was carried out through a population matching cohort analysis.

The findings are in line with previous studies and the additional deeper analysis of gender and MS provides valuable insight into this important topic.

The introduction provided good background to published studies on gender inequalities, the impact of earnings following a diagnosis of MS and the theorised reasons for these variations.

The methods are robust and the authors have used additional techniques to allow for some of the limitations of the data (ie using Huber's).

There are a number of different results to consider due to the main effect and interactions factorial results. These are well explained by the authors and the final section of the results does a great job at summarising a complex analysis.

The discussion is well written and insightful. I particularly felt it was important to discuss the impact of part time hours and the author's discussion of methods used to try and limit this (through SA/DP) is welcomed.

Overall I found this paper very useful and enjoyed reading it. I have set out one minor comment for the authors consideration below:

Abstract/Introduction: (Line 49 and Line 104) The authors are looking at a number of relationships for earnings in the paper, I wonder if it would be helpful to the reader to have the aims of the study to be even more explicit. E.g. that it is looking at gender differences between those diagnosed MS and those without MS AND between those diagnosed while also considering family status and occupation.

**Authors response:** Thank you for your comments and the opportunity to further clarify the aims of the study. We have now stated explicitly that differences are observed between those with and without MS while considering type of occupation and family composition among PwMS. (See: Page 2; lines 49-50; and Page 4, lines 104-105).
